# Physical activity and sleep quality among college students: the sequential indirect associations of negative attentional bias and bedtime procrastination

**DOI:** 10.3389/fpsyg.2026.1910032

**Published:** 2026-10-28

**Authors:** Jiameng Sun, Rui Li, Jie Wu, Qiqi Zhou, Jinrui Feng, Mohamad Nizam Nazarudin

**Affiliations:** 1 Institute of Physical Education, Nantong Normal College, Nantong, China; 2 Faculty of Education, Universiti Kebangsaan Malaysia, Bangi, Selangor, Malaysia; 3 Institute of Sports Science, Nantong University, Nantong, China; 4 Nantong Health Vocational College, Nantong, China; 5 Department of Physical Education, Shanghai University of Traditional Chinese Medicine, Shanghai, China

**Keywords:** bedtime procrastination, college students, negative attentional bias, physical activity, sequential association model, sleep quality

## Abstract

Sleep quality is fundamental to college students’ physical and psychological health and academic functioning. Amid increasing digitalization and stress, sleep problems remain prevalent among college students. Previous research has shown that physical activity, cognitive processing styles, and behavioral regulation patterns are associated with sleep outcomes, but these variables have often been examined separately, with limited attention to their integrated association structure. Based on cross-sectional survey data, this study used latent-variable structural equation modeling to examine the sequential indirect association pattern among physical activity, negative attentional bias, bedtime procrastination, and sleep quality. Using cluster sampling, data were collected from 921 college students from four universities in East China (439 males, 482 females; M_age = 20.34, SD = 1.59). Measures included the Physical Activity Rating Scale (PARS-3), Negative Attentional Bias Scale, Bedtime Procrastination Scale, and Pittsburgh Sleep Quality Index (PSQI). Structural equation modeling and bootstrap methods examined indirect associations. Results indicated that physical activity was significantly associated with lower PSQI scores, indicating better sleep quality (B_total = −0.072, *β*_total = −0.440, *p* < 0.001). Negative attentional bias and bedtime procrastination both showed significant partial indirect associations. The simple indirect associations through negative attentional bias and bedtime procrastination accounted for 20.8 and 15.3% of the total association, respectively. The sequential indirect association “physical activity → negative attentional bias → bedtime procrastination → sleep quality” was also significant, although its proportion was relatively small (4.2%). Overall, the total indirect association accounted for 40.3% of the total association. Multi-group structural analysis indicated that the association structure was broadly comparable across gender groups, although associations involving bedtime procrastination appeared stronger among female students. By integrating cognitive processing and behavioral regulation perspectives, this study provides a more integrative view of the association between physical activity and sleep quality. The findings suggest that college students’ sleep problems may be embedded within a broader system of health behaviors and psychological processes. Given the cross-sectional design, future longitudinal or experimental studies should examine the dynamic associations among these variables.

## Introduction

Sleep quality is a fundamental basis for maintaining cognitive functioning, emotional stability, and physical health. Previous studies have shown that insufficient sleep or poor sleep quality is closely associated with reduced attention, impaired executive function, lower academic performance, and an increased risk of anxiety and depression (Benitez and Gunstad, 2012; Hyndych et al., 2025). In contrast, adequate sleep contributes to more effective emotion regulation, improved information integration, and better recovery from stress (Sharma, 2026). During the college years, individuals are in a critical developmental period characterized by ongoing growth in self-regulation and transitions in social roles. At this stage, students often have greater behavioral autonomy but also face multiple sources of pressure, including academic competition, examination demands, coursework deadlines, performance evaluation, uncertainty about future employment, and challenges in social adjustment (Nuralieva et al., 2023). However, epidemiological evidence indicates that sleep problems are highly prevalent among college students and have shown a persistent upward trend (Zhou et al., 2022). Factors such as prolonged electronic device use, irregular daily routines, academic stress, and increased perceived stress have all been linked to declining sleep quality in this population (Oliveira et al., 2026). Given that sleep quality during the college years may have cumulative associations with long-term mental health trajectories, examining modifiable factors associated with sleep quality is of both practical and theoretical importance.

Previous research on improving sleep quality has primarily focused on approaches such as cognitive–behavioral interventions, sleep hygiene education, and pharmacological treatments. Although these methods can be effective in clinical or experimental settings, their broader implementation is often limited by factors such as cost, adherence difficulties, or potential side effects (Valentino and Volkow, 2020). In comparison, physical activity has increasingly attracted attention as a sustainable and low-cost behavior with broad health benefits. A substantial body of research has shown that regular physical activity is linked to shorter sleep onset latency, higher sleep efficiency, and better subjective sleep quality (Semplonius and Willoughby, 2018; Maruszczak et al., 2025). From a physiological perspective, physical activity may be associated with sleep through the regulation of circadian rhythms, the strengthening of sleep homeostasis, and the improvement of autonomic nervous system balance (Canonichesi et al., 2025; Zhu et al., 2025). From a psychological perspective, physical activity has been consistently linked to reduced stress, improved mood, and enhanced executive control (Xu et al., 2025; Wang et al., 2025). However, most existing studies have mainly examined stress or emotional factors as potential explanatory variables, while relatively little attention has been given to cognitive biases at the level of information processing. In recent years, integrative research has increasingly emphasized multi-step psychological association patterns that integrate emotional, cognitive, and behavioral processes within a unified framework. For example, previous research has suggested that psychological resilience and emotional distress may form sequential indirect association patterns linking physical activity to behavioral outcomes (Yan et al., 2026). Similarly, recent studies have shown that early adverse experiences may be associated with health behaviors through multi-step emotional pathways, including depression, anxiety, and subjective well-being (Yan et al., 2026). These findings suggest that health-related outcomes may reflect the combined influence of multiple psychological processes rather than single factors. However, relatively few studies have extended such integrative modeling approaches to sleep-related processes, particularly by simultaneously incorporating cognitive processing (e.g., attentional bias) and behavioral regulation (e.g., bedtime procrastination).

In college student populations in particular, sleep difficulties are often accompanied by elevated cognitive arousal before sleep. Therefore, it is necessary to further explore the association between physical activity and sleep quality from the perspective of cognitive processing. In this context, beyond its well-established physiological benefits, physical activity can also be conceptualized as a regulatory resource that operates across multiple psychological domains. From the perspective of the stress-buffering hypothesis (Cohen and Wills, 1985), engagement in physical activity may attenuate the impact of both environmental and internal stressors by enhancing individuals’ adaptive capacity. At the same time, self-regulation theory (Baumeister et al., 2007) suggests that sustained participation in physical activity reflects and reinforces individuals’ regulatory abilities, including goal-directed behavior, impulse control, and behavioral consistency. In addition, emotion regulation frameworks (Gross, 2015) indicate that physical activity is associated with reduced negative affect and improved emotional stability (Lubans et al., 2016), which may in turn be associated with both cognitive processing and behavioral responses. Within this integrative framework, sleep-related processes may be understood as the outcome of coordinated functioning across multiple levels of psychological regulation. In particular, negative attentional bias and bedtime procrastination can be conceptualized as manifestations of regulatory processes at different levels. Negative attentional bias reflects a tendency to preferentially process negative information, which has been linked to sustained cognitive arousal and emotional activation (Bar-Haim et al., 2007; Pool et al., 2016; Kaiser et al., 2018). In contrast, bedtime procrastination refers to voluntarily delaying bedtime in the absence of external constraints and is commonly understood as a behavioral manifestation of self-regulation failure (Kroese et al., 2016; Steel, 2007). Building on these perspectives, physical activity may be associated with sleep quality through a multi-level pattern of associations. Specifically, higher levels of physical activity may be linked to more adaptive emotion regulation and reduced cognitive bias toward negative information, which may in turn be associated with a lower likelihood of maladaptive behavioral patterns such as bedtime procrastination. Within this cognitive–behavioral association pattern, physical activity may be indirectly associated with sleep-related outcomes. Taken together, physical activity, cognitive bias, and behavioral regulation may be better understood as interconnected processes underlying individual differences in sleep quality. Among these processes, cognitive-level factors may represent an important level at which such regulatory associations are reflected.

Negative attentional bias is particularly relevant to sleep-related processes because difficulty disengaging from negative or threatening information may prolong cognitive and emotional activation (Bar-Haim et al., 2007). This bias has been widely documented in research on anxiety and emotional disorders and is closely linked to sustained cognitive and emotional activation (Pool et al., 2016). Individuals with higher levels of negative attentional bias often find it difficult to disengage from negative information and may remain in a state of repeated cognitive activation (Kaiser et al., 2018). Previous studies have shown that sleep problems are significantly associated with elevated levels of cognitive arousal before bedtime, and a tendency to process negative information may be associated with higher levels of such cognitive arousal (Palagini et al., 2017; Gobin et al., 2015). At the same time, neurocognitive research suggests that regular physical activity is linked to enhanced functioning of executive control networks, which may be related to the regulation of attentional allocation and bias (Wang et al., 2023). Based on this perspective, individuals with higher levels of physical activity may report lower levels of negative attentional bias, which may also be indirectly associated with sleep quality. However, studies that integrate physical activity, attentional bias, and sleep quality within a single analytical framework remain relatively limited.

In addition to cognitive processing characteristics, behavioral self-regulation is also an important factor associated with sleep. Bedtime procrastination refers to the behavior of voluntarily delaying bedtime in the absence of external constraints (Kroese et al., 2016). According to self-regulation theory and dual-system models (Steel, 2007; Grund et al., 2018), when immediate emotion regulation needs or short-term reward motivations dominate, individuals may place less emphasis on long-term health goals, which may be associated with procrastination behaviors. Previous research has shown that bedtime procrastination is significantly associated with insufficient sleep duration, lower sleep efficiency, and poorer subjective sleep quality (Zhu et al., 2022; Zhang and Wu, 2020). From the perspective of a cognitive–behavioral continuum, patterns of information processing may be closely related to problems in behavioral regulation. When individuals report higher levels of negative attentional bias, their sustained attention to negative stimuli may be associated with greater emotional burden and a higher tendency toward avoidant or compensatory behaviors that delay sleep. In this sense, negative attentional bias may be associated with bedtime procrastination and may further relate to sleep quality. However, relatively few studies have examined these two factors simultaneously as sequential patterns of associations linking physical activity and sleep quality.

Based on the above background, the present study focused on college students and examined the association between physical activity and sleep quality, with particular attention to the potential sequential indirect associations of negative attentional bias and bedtime procrastination. Specifically, this study aimed to: (1) examine the association between physical activity and sleep quality; (2) test whether negative attentional bias and bedtime procrastination separately account for indirect associations between physical activity and sleep quality; and (3) further explore whether these two factors form a sequential indirect association pattern linking physical activity and sleep quality. By integrating variables related to cognitive processing and behavioral regulation, this study seeks to provide more systematic evidence for understanding the psychological structure underlying individual differences in sleep quality among college students. Based on the theoretical background and previous empirical findings, the following hypotheses were proposed:

*H1a*: Physical activity would be negatively associated with PSQI scores, indicating that higher physical activity would be associated with better sleep quality.

*H1b*: Physical activity would be negatively associated with negative attentional bias and bedtime procrastination, whereas negative attentional bias and bedtime procrastination would be positively associated with PSQI scores.

*H2*: Negative attentional bias and bedtime procrastination would show significant simple indirect associations in the relationship between physical activity and PSQI-assessed sleep quality.

*H3*: Negative attentional bias and bedtime procrastination would form a sequential indirect association pattern linking physical activity and PSQI-assessed sleep quality.

In addition, gender differences in the strength of these structural associations were examined exploratorily using multi-group SEM.

## Methods

### Ethical approval and informed consent

This study was approved by the Ethics Committee of Nantong University (Approval No.: TD-2024-109). Prior to data collection, all participants were informed of the study purpose, procedures, and relevant considerations. Informed consent was obtained electronically, with participants required to indicate their agreement by selecting the option “agree to participate” before completing the questionnaire. Participation was entirely voluntary, and participants were free to withdraw at any stage without any consequences. All procedures performed in this study involving human participants were in accordance with the ethical standards of the institutional research committee and with the 1964 Helsinki Declaration and its later amendments or comparable ethical standards.

### Data source

The data for this study were collected from undergraduate students at four universities in East China, with participants recruited through class-based cluster sampling within each university. Cluster sampling was employed, with classes serving as the primary sampling units and universities treated as study sites. Under the coordination of the research team and the participating universities, questionnaires were administered in organized group settings. All selected classes that met the inclusion criteria were invited to participate. Data were collected between September 2025 and November 2025 using an online questionnaire generated through the Wenjuanxing platform (https://www.wjx.cn/). Although the questionnaire was administered electronically, participants completed it in person during organized sessions on campus. During data collection, trained researchers provided standardized instructions to ensure consistency in the testing environment and administration procedures. A total of 950 questionnaires were obtained. The screening criteria included: (1) incomplete questionnaires; (2) responses showing clear signs of invalid answering behavior (e.g., extremely short completion time); and (3) cases with substantial missing data on key variables that could not be included in statistical analyses. After screening, 921 valid responses were retained for the final analysis, including 439 males and 482 females. The mean age of the participants was 20.34 years (SD = 1.59).

### Measures

#### Physical activity

Physical activity levels were assessed using the Physical Activity Rating Scale–3 (PARS-3) developed by D. C. Liang (Liang, 1994). This scale has been widely used in Chinese populations and has demonstrated good reliability and validity. The PARS-3 measures physical activity across three dimensions: exercise frequency, exercise duration, and exercise intensity. The scale consists of three items, each rated on a five-point Likert scale (1–5). The total physical activity score was calculated using the following formula: Physical Activity Score = Frequency × (Duration − 1) × Intensity. The theoretical score ranges from 0 to 100. Based on the total score, physical activity levels were categorized into three groups: low (≤19), moderate (20–42), and high (≥43). According to the standard scoring criteria of the PARS-3, total scores are categorized into low, moderate, and high levels of physical activity. This classification approach has been widely used in studies involving Chinese populations and provides a practical framework for interpreting individual differences in physical activity levels, although the brevity and self-report nature of the instrument should be considered when interpreting the findings (Wang et al., 2025).

#### Sleep quality

Sleep quality was assessed using the Pittsburgh Sleep Quality Index (PSQI). The PSQI was originally developed by Buysse and colleagues at the University of Pittsburgh. In this study, the Chinese version translated and adapted by Liu Xianchen and colleagues was used to evaluate participants’ sleep quality over the past month (Liu et al., 1996). The PSQI includes 19 self-rated items and 5 items rated by others. Among these, 18 self-rated items contribute to the scoring and form seven components: subjective sleep quality (1 item), sleep latency (2 items), sleep duration (1 item), sleep efficiency (3 items), sleep disturbances (9 items), use of sleep medication (1 item), and daytime dysfunction (2 items). Each component is scored on a four-point scale (0–3). The total PSQI score ranges from 0 to 21, with higher scores indicating poorer sleep quality. In the present study, PSQI total scores were treated as a continuous variable in all analyses, and the PSQI showed good internal consistency in the present sample, with Cronbach’s α = 0.897.

#### Negative attentional bias

Negative attentional bias was measured using the Negative Attentional Bias subscale of the Positive/Negative Attentional Bias Scale developed by Noguchi and colleagues (Noguchi et al., 2006). The Chinese version used in this study was revised by Lü et al. (Lü et al., 2016). The subscale consists of 11 items, each rated on a five-point Likert scale (1 = strongly disagree to 5 = strongly agree). All items are scored in the same direction, and mean item scores were used in the analyses, with higher scores indicating a greater tendency toward negative attentional bias. In the present study, the internal consistency of the scale was high, with Cronbach’s α = 0.934.

#### Bedtime procrastination

Bedtime procrastination was measured using the Bedtime Procrastination Scale developed by Kroese et al. (Kroese et al., 2016). The Chinese version revised by Zhang Lu and colleagues was used in this study (Zhang et al., 2021). The scale consists of nine items, rated on a five-point Likert scale (1 = never to 5 = always). Among them, four items are reverse-coded. Mean item scores were used in the analyses, with higher scores indicating a greater tendency toward bedtime procrastination. In the present study, the scale showed good internal consistency, with Cronbach’s α = 0.928.

### Statistical analysis

All statistical analyses were conducted using SPSS 26.0 and AMOS 26.0. The primary analysis was conducted using latent-variable structural equation modeling, with negative attentional bias and bedtime procrastination specified as latent constructs and physical activity and PSQI-assessed sleep quality entered as observed composite variables. Descriptive statistics were first calculated for physical activity, negative attentional bias, bedtime procrastination, and sleep quality, including the mean, standard deviation, skewness, and kurtosis, and the distributional characteristics of the variables were further examined using Shapiro–Wilk tests, Q-Q plot inspection, and Mardia’s test of multivariate normality. Confirmatory factor analysis (CFA) was performed using AMOS 26.0 to assess the measurement structure and discriminant validity of the two multi-item latent constructs, namely negative attentional bias and bedtime procrastination. Model parameters were estimated using the maximum likelihood method, and the assumptions relevant to this estimator were evaluated using descriptive distributional indices, inferential normality tests, Q-Q plot inspection, and Mardia’s test of multivariate normality. Model fit was evaluated using commonly reported indices, including the chi-square to degrees of freedom ratio (*χ*^2^/df), comparative fit index (CFI), Tucker–Lewis index (TLI), root mean square error of approximation (RMSEA), and standardized root mean square residual (SRMR). In general, values of *χ*^2^/df below 5, CFI and TLI above 0.90, and RMSEA and SRMR below 0.08 were considered indicative of acceptable model fit. Pearson correlation analysis was used to assess the bivariate associations among the continuous study variables. The distribution of poor sleep quality was further described using commonly adopted PSQI screening cut-off criteria (PSQI > 5 and PSQI > 7). Multicollinearity diagnostics were conducted for the main predictors using variance inflation factors (VIFs). Subsequently, a latent-variable SEM was conducted to examine the sequential indirect associations of negative attentional bias and bedtime procrastination in the association between physical activity and PSQI-assessed sleep quality. To obtain robust estimates of the indirect associations, the bias-corrected bootstrap method with 5,000 resamples was applied to generate 95% confidence intervals (CIs). An indirect association was considered statistically significant when the confidence interval did not include zero. Statistical significance was set at *p* < 0.05. For gender comparisons, Cohen’s d was calculated as an effect size for independent-samples t-tests, and *R*^2^ was reported for endogenous variables in the SEM model. All SEM-based results were interpreted as statistical associations rather than causal pathways because of the cross-sectional design.

## Results

### Common method bias and normality test

Descriptive statistics for the main variables are presented in Table 1. The mean score for physical activity was 31.275 (SD = 29.784), and the mean score for the Pittsburgh Sleep Quality Index was 6.569 (SD = 4.895). The mean scores for negative attentional bias and bedtime procrastination were 2.074 (SD = 0.734) and 2.091 (SD = 0.736), respectively. The skewness values ranged from 0.590 to 0.850, and the kurtosis values ranged from −0.386 to −0.032. All skewness and kurtosis values were within the commonly accepted threshold of ±2, suggesting no substantial univariate deviation from normality based on descriptive criteria. Shapiro–Wilk tests were statistically significant for physical activity (W = 0.879, *p* < 0.001), PSQI scores (W = 0.920, *p* < 0.001), negative attentional bias (W = 0.954, *p* < 0.001), and bedtime procrastination (W = 0.951, *p* < 0.001). Mardia’s test also indicated multivariate non-normality. Given the large sample size, these results were interpreted together with skewness, kurtosis, Q-Q plot inspection, and bootstrap confidence intervals.

**Table 1:** Descriptive statistics and normality tests of the study variables (*N* = 921).

Variable	Max	Min	Mean	SD	Skewness	Kurtosis	*W*	*P*
Physical activity	100.000	0.000	31.275	29.784	0.827	−0.386	0.879	< 0.001
Sleep quality (PSQI)	20.000	0.000	6.569	4.895	0.850	−0.032	0.920	< 0.001
Negative attentional bias	4.000	1.000	2.074	0.734	0.590	−0.254	0.954	< 0.001
Bedtime procrastination	4.000	1.000	2.091	0.736	0.626	−0.079	0.951	< 0.001

For negative attentional bias and bedtime procrastination, mean item scores were used; therefore, the minimum and maximum values represent observed sample ranges rather than theoretical scale ranges. W = Shapiro–Wilk test statistic.

Based on commonly adopted PSQI screening cut-off criteria, 449 participants (48.8%) had PSQI scores greater than 5, and 324 participants (35.2%) had PSQI scores greater than 7. These results indicate that poor sleep quality was relatively common in the present sample. The cut-off results were used descriptively, whereas PSQI total scores were treated as continuous variables in the main analyses. To assess the potential presence of common method bias, given that all data were collected through self-report questionnaires, Harman’s single-factor test was conducted. An unrotated principal component analysis was performed on all measurement items. The analysis extracted several factors with eigenvalues greater than 1. The first factor accounted for 37.089% of the total variance, which is below the commonly used 40% threshold in behavioral research (Podsakoff et al., 2003). In addition, multiple factors jointly explained a substantial proportion of the total variance, with no single factor dominating the variance structure. These results suggest that serious common method bias is unlikely to be present in the data, although this possibility cannot be completely ruled out because all variables were measured using self-report questionnaires.

### Gender differences

To examine whether the study variables differed by gender, independent-samples t-tests were conducted, and Cohen’s d was calculated to evaluate the magnitude of gender differences. The results are presented in Table 2.

**Table 2:** Gender differences and effect sizes in the study variables.

Variable	Male (M ± SD)	Female (M ± SD)	*t*	*p*	Cohen’s *d*
Physical activity	32.62 ± 30.14	30.05 ± 29.43	1.304	0.193	0.086
Sleep quality (PSQI)	5.97 ± 4.56	7.11 ± 5.12	−3.564	< 0.001	−0.235
Negative attentional bias	2.01 ± 0.71	2.13 ± 0.75	−2.524	0.012	−0.167
Bedtime procrastination	2.01 ± 0.70	2.17 ± 0.76	−3.268	0.001	−0.216

df, 919 for all independent-samples t-tests. Cohen’s d was calculated as the standardized mean difference between male and female students.

No significant gender difference was observed in physical activity levels between male and female college students (t(919) = 1.304, *p* = 0.193, Cohen’s *d* = 0.086). In contrast, sleep quality differed significantly by gender (t(919) = −3.564, *p* < 0.001, Cohen’s *d* = −0.235), with female students reporting higher PSQI scores, indicating relatively poorer sleep quality. A significant gender difference was also observed for negative attentional bias (t(919) = −2.524, *p* = 0.012, Cohen’s *d* = −0.167), with female students scoring higher than male students. Similarly, bedtime procrastination differed significantly by gender (t(919) = −3.268, *p* = 0.001, Cohen’s *d* = −0.216), with females reporting higher levels. Although these gender differences were statistically significant, the corresponding effect sizes were small (|d| = 0.167–0.235), suggesting that the magnitude of these differences should be interpreted cautiously.

### Age differences

To examine whether age was associated with the continuous study variables, Pearson correlation analysis was conducted. The results are reported in the text rather than in a separate table to improve readability.

Age was not significantly correlated with physical activity (*r* = 0.026, *p* = 0.435), PSQI scores (*r* = 0.011, *p* = 0.734), negative attentional bias (*r* = −0.019, *p* = 0.561), or bedtime procrastination (*r* = 0.020, *p* = 0.538). These results suggest that age was not significantly associated with the main continuous variables in the present sample.

### Confirmatory factor analysis

To assess discriminant validity between the two multi-item psychological constructs, namely negative attentional bias and bedtime procrastination, a two-factor model and a single-factor model were compared. In the two-factor model, negative attentional bias items and bedtime procrastination items were loaded onto their respective latent variables, whereas in the single-factor model, all items from these two scales were loaded onto a single latent factor. Model fit indices are reported in Table 3.

**Table 3:** Fit indices for the competing measurement models.

Model	*χ* ^2^	df	*χ*^2^/df	RMSEA	CFI	TLI	SRMR
Two-factor model	753.343	169	4.458	0.061	0.950	0.943	0.045
Single-factor model	5239.429	170	30.820	0.180	0.563	0.511	0.218

The results showed that the two-factor model demonstrated an acceptable fit to the data (*χ*^2^ = 753.343, df = 169, *χ*^2^/df = 4.458, RMSEA = 0.061, CFI = 0.950, TLI = 0.943, SRMR = 0.045). In contrast, the single-factor model showed poor fit (*χ*^2^ = 5239.429, df = 170, *χ*^2^/df = 30.820, RMSEA = 0.180, CFI = 0.563, TLI = 0.511, SRMR = 0.218), with all fit indices failing to reach recommended thresholds. Overall, the fit indices indicated that the two-factor model fit the data substantially better than the single-factor model, suggesting that distinguishing negative attentional bias and bedtime procrastination as two latent constructs is more consistent with the observed data. These findings support discriminant validity between negative attentional bias and bedtime procrastination and indicate that the variance in the measurement items from these two scales was not dominated by a single latent factor. It should be noted that the two-factor and single-factor models were specified as simplified structures for methodological purposes rather than to represent the full theoretical framework of the study. Specifically, these models were used to assess discriminant validity among the constructs and to examine the potential influence of common method bias through comparison with a single-factor model. Therefore, this analysis was not intended to test a full four-construct latent measurement model, but rather to provide a focused evaluation of the measurement structure of the two multi-item psychological constructs.

### Correlation analysis and discriminant validity

The means, standard deviations, correlation coefficients among the continuous study variables, and the square roots of the average variance extracted (AVE) for the two multi-item psychological constructs are presented in Table 4. Physical activity was significantly negatively correlated with Pittsburgh Sleep Quality Index (PSQI) scores (*r* = −0.440, *p* < 0.001), as well as with negative attentional bias (*r* = −0.338, *p* < 0.001) and bedtime procrastination (*r* = −0.327, *p* < 0.001). Considering that higher PSQI scores indicate poorer sleep quality, these results indicate that higher levels of physical activity correspond to better sleep quality, lower negative attentional bias, and lower levels of bedtime procrastination. Regarding the indirect association variables, negative attentional bias was positively correlated with PSQI scores (*r* = 0.422, *p* < 0.001), and bedtime procrastination was also positively correlated with PSQI scores (*r* = 0.407, *p* < 0.001). In addition, negative attentional bias was positively correlated with bedtime procrastination (*r* = 0.281, *p* < 0.001). These correlation patterns provide statistical support for subsequent model testing. Because gender differences were examined using independent-samples t-tests, gender was not included in the correlation table to avoid redundant point-biserial correlation results. In contrast, age was not significantly correlated with any of the main continuous variables.

**Table 4:** Means, standard deviations, correlations, and square roots of AVE for the continuous study variables.

Variable	M ± SD	1	2	3	4
1. Physical activity	31.27 ± 29.78	1			
2. Sleep quality (PSQI)	6.57 ± 4.89	−0.440^***^	1.000		
3. Negative attentional bias	2.07 ± 0.73	−0.338^***^	0.422^***^	√AVE = 0.750	
4. Bedtime procrastination	2.09 ± 0.74	−0.327^***^	0.407^***^	0.281^***^	√AVE = 0.769

Higher PSQI scores indicate poorer sleep quality. Values on the diagonal represent the square roots of the average variance extracted (AVE) for the two multi-item latent constructs. Because physical activity and PSQI scores were treated as observed composite variables, AVE values were not estimated for these variables. ***p < 0.001.

Multicollinearity diagnostics showed that the VIF values were 1.208 for physical activity, 1.172 for negative attentional bias, and 1.162 for bedtime procrastination, indicating no serious multicollinearity among the main predictors. Discriminant validity was further assessed using the Fornell–Larcker criterion by comparing the square roots of the AVE values for each latent construct with the inter-construct correlations. The square roots of the AVE for negative attentional bias (√AVE = 0.750) and bedtime procrastination (√AVE = 0.769) were greater than their correlations with other constructs, indicating adequate discriminant validity. Overall, the correlation patterns were consistent with theoretical expectations, and discriminant validity between negative attentional bias and bedtime procrastination met acceptable standards. Age was examined as a demographic covariate, whereas gender was examined through both independent-samples t-tests and multi-group SEM.

### Sequential indirect association analysis

To examine the sequential indirect associations of negative attentional bias and bedtime procrastination in the association between physical activity and PSQI-assessed sleep quality, latent-variable structural equation modeling was conducted. The results are reported in Table 5 and Figure 1.

**Table 5:** Structural path coefficients in the latent-variable SEM model.

Path	B	SE	*β*	*p*
Physical activity → Negative attentional bias	−0.008	0.001	−0.346	< 0.001
Physical activity → Bedtime procrastination	−0.007	0.001	−0.270	< 0.001
Negative attentional bias → Bedtime procrastination	0.212	0.039	0.196	< 0.001
Physical activity → PSQI scores	−0.043	0.005	−0.263	< 0.001
Negative attentional bias → PSQI scores	1.941	0.229	0.270	< 0.001
Bedtime procrastination → PSQI scores	1.654	0.207	0.249	< 0.001

B, unstandardized coefficient; *β*, standardized coefficient; SE, standard error; PSQI, Pittsburgh Sleep Quality Index. Higher PSQI scores indicate poorer sleep quality. *R*^2^ values were 0.120 for negative attentional bias, 0.148 for bedtime procrastination, and 0.336 for PSQI scores.

**Figure 1:**
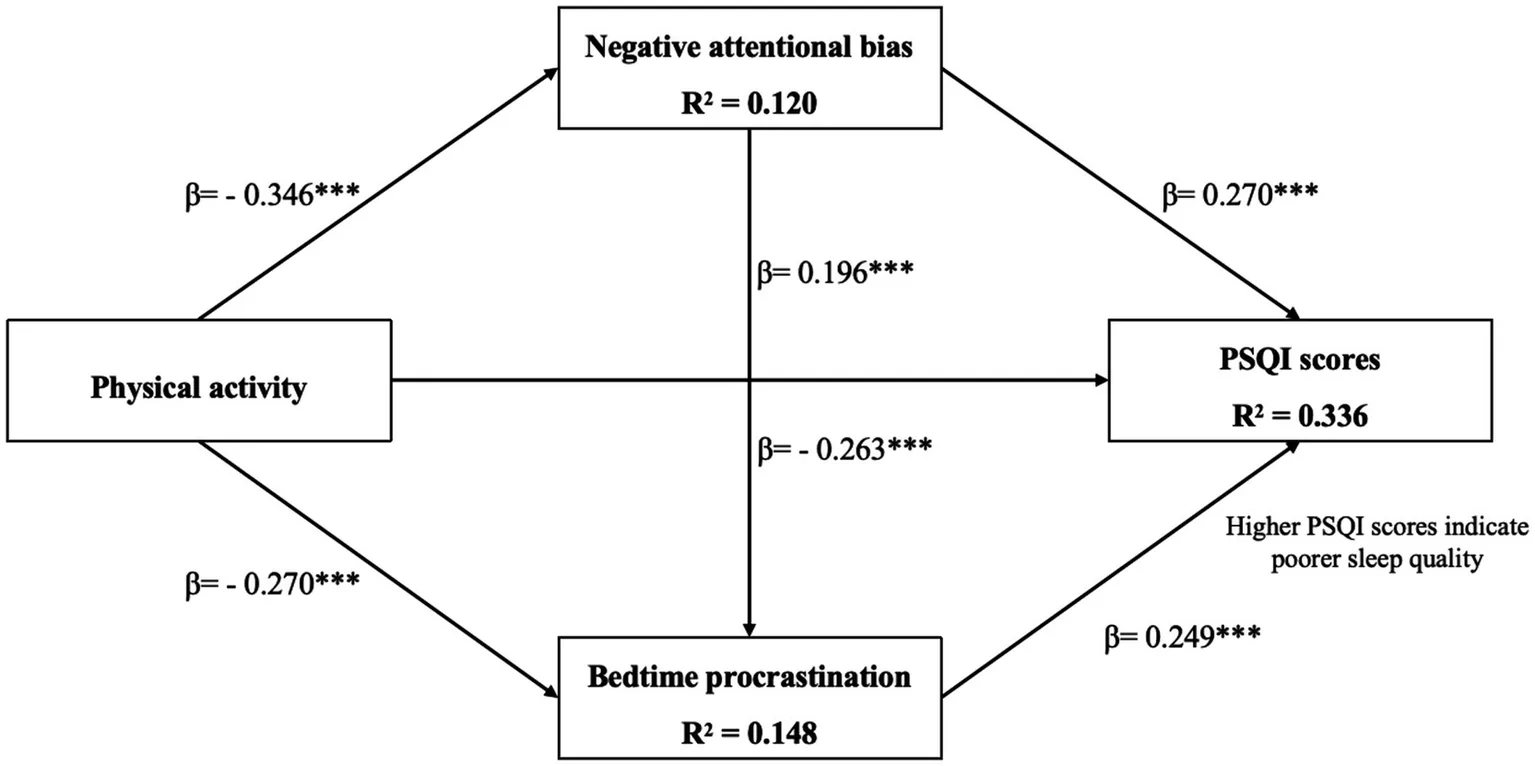
Latent-variable structural equation model linking physical activity, negative attentional bias, bedtime procrastination, and PSQI scores.

In the latent-variable SEM model, physical activity was significantly negatively associated with negative attentional bias (*B* = −0.008, *β* = −0.346, *p* < 0.001) and bedtime procrastination (*B* = −0.007, *β* = −0.270, *p* < 0.001). Negative attentional bias was significantly positively associated with bedtime procrastination (*B* = 0.212, *β* = 0.196, *p* < 0.001). Physical activity was also significantly negatively associated with PSQI scores (*B* = −0.043, *β* = −0.263, *p* < 0.001), indicating that higher levels of physical activity were associated with better sleep quality. Meanwhile, negative attentional bias (*B* = 1.941, *β* = 0.270, *p* < 0.001) and bedtime procrastination (*B* = 1.654, *β* = 0.249, *p* < 0.001) were both positively associated with PSQI scores. The SEM model explained 12.0% of the variance in negative attentional bias (*R*^2^ = 0.120), 14.8% of the variance in bedtime procrastination (*R*^2^ = 0.148), and 33.6% of the variance in PSQI scores (*R*^2^ = 0.336). All structural coefficients in the latent-variable SEM model were statistically significant at *p* < 0.001.

Overall, the results indicate that physical activity is directly associated with PSQI-assessed sleep quality and also indirectly associated with PSQI-assessed sleep quality through negative attentional bias and bedtime procrastination. After including these variables in the model, the direct association remained significant, indicating a partial indirect association pattern.

### Bootstrap test of sequential indirect associations

To further examine the sequential indirect associations of negative attentional bias and bedtime procrastination in the association between physical activity and PSQI-assessed sleep quality, a bias-corrected bootstrap method was applied. The results are reported in Table 6.

**Table 6:** Bootstrap estimates of direct, indirect, and total associations in the latent-variable SEM model.

Category	Path	*B*	95% CI for B	*p*	Proportion
Direct association	Physical activity → PSQI scores	−0.043	[−0.054, −0.032]	< 0.001	59.70%
Simple indirect association	Physical activity → Negative attentional bias → PSQI scores	−0.015	[−0.021, −0.011]	< 0.001	20.80%
	Physical activity → Bedtime procrastination → PSQI scores	−0.011	[−0.016, −0.007]	< 0.001	15.30%
Sequential indirect association	Physical activity → Negative attentional bias → Bedtime procrastination → PSQI scores	−0.003	[−0.005, −0.001]	< 0.001	4.20%
Total indirect association	Physical activity → … → PSQI scores	−0.029	[−0.036, −0.023]	< 0.001	40.30%
Total association	Physical activity → PSQI scores	−0.072	[−0.082, −0.063]	< 0.001	—

B, unstandardized bootstrap estimate; CI, confidence interval; PSQI, Pittsburgh Sleep Quality Index. Higher PSQI scores indicate poorer sleep quality. Proportions were calculated relative to the total association.

First, regarding the direct association, physical activity showed a significant direct association with lower PSQI scores (*B* = −0.043, *β* = −0.263, 95% CI for B [−0.054, −0.032], *p* < 0.001). In addition, negative attentional bias (*B* = 1.941, *β* = 0.270, *p* < 0.001) and bedtime procrastination (*B* = 1.654, *β* = 0.249, *p* < 0.001) were both positively associated with PSQI scores. Physical activity was also negatively associated with negative attentional bias (*B* = −0.008, *β* = −0.346, *p* < 0.001) and bedtime procrastination (*B* = −0.007, *β* = −0.270, *p* < 0.001), whereas negative attentional bias was positively associated with bedtime procrastination (*B* = 0.212, *β* = 0.196, *p* < 0.001). Regarding the simple indirect associations, physical activity was indirectly associated with PSQI scores through negative attentional bias (*B* = −0.015, 95% CI [−0.021, −0.011], *p* < 0.001), accounting for 20.8% of the total association. The indirect association through bedtime procrastination was also significant (*B* = −0.011, 95% CI [−0.016, −0.007], *p* < 0.001), accounting for 15.3% of the total association. For the sequential association pattern, physical activity was indirectly associated with PSQI scores through the pathway of negative attentional bias → bedtime procrastination (*B* = −0.003, 95% CI [−0.005, −0.001], *p* < 0.001), which accounted for only 4.2% of the total association.

Overall, the total indirect association was significant (*B* = −0.029, *β* = −0.178, 95% CI for B [−0.036, −0.023], *p* < 0.001), accounting for 40.3% of the total association. The total association between physical activity and PSQI scores was also significant (*B* = −0.072, *β* = −0.440, 95% CI for B [−0.082, −0.063], *p* < 0.001). All reported direct, indirect, and total associations were statistically significant at *p* < 0.001. Because the direct association between physical activity and PSQI-assessed sleep quality remained significant after including the other variables, the results indicate a partial indirect association pattern involving negative attentional bias and bedtime procrastination.

### Multi-group structural equation modeling analysis (gender differences)

To examine whether the structural associations were comparable across gender groups, a multi-group structural equation modeling (SEM) analysis was conducted. The results are reported in Table 7, and model equivalence was further evaluated using changes in CFI and RMSEA. Before comparing structural associations across gender groups, measurement equivalence of the two latent constructs was examined using configural, metric, and scalar invariance models. The configural model showed acceptable fit (*χ*^2^ = 943.476, df = 338, CFI = 0.948, RMSEA = 0.044). Compared with the configural model, the metric invariance model showed minimal change in fit (*χ*^2^ = 982.125, df = 356, CFI = 0.946, RMSEA = 0.044; ΔCFI = 0.002, ΔRMSEA = 0.000). The scalar invariance model also showed acceptable change in fit compared with the metric model (*χ*^2^ = 1012.018, df = 374, CFI = 0.945, RMSEA = 0.043; ΔCFI = 0.001, ΔRMSEA = 0.001). These results indicate that the measurement structure was sufficiently comparable across gender groups. Therefore, subsequent multi-group SEM analyses were conducted to examine whether structural path coefficients differed by gender. The association between physical activity and PSQI scores was significant for both male and female students (males: *B* = −0.041, *β* = −0.271, *p* < 0.001; females: *B* = −0.046, *β* = −0.266, *p* < 0.001), with no significant difference between groups (critical ratio = 0.505, *p* = 0.614). Similarly, the association between negative attentional bias and PSQI scores was significant in both groups (males: *B* = 2.021, *β* = 0.302, *p* < 0.001; females: *B* = 1.706, *β* = 0.226, *p* < 0.001), and the difference between genders was not significant (critical ratio = 0.696, *p* = 0.487). A significant gender difference was observed in the association between bedtime procrastination and PSQI scores (critical ratio = −3.719, *p* < 0.001), with the association appearing stronger among female students (*B* = 2.321, *β* = 0.341, *p* < 0.001) than among male students (*B* = 0.788, *β* = 0.123, *p* = 0.007). In addition, the association between negative attentional bias and bedtime procrastination was significant among female students (*B* = 0.290, *β* = 0.262, *p* < 0.001), but not among male students (*B* = 0.102, *β* = 0.097, *p* = 0.060), and the between-group difference was statistically significant (critical ratio = −2.417, *p* = 0.016). The remaining associations, physical activity → negative attentional bias and physical activity → bedtime procrastination, were significant in both gender groups, with no significant differences between males and females.

**Table 7:** Gender-specific structural path coefficients in the multi-group SEM model.

Path	Male					Female					Group C.R.	Group *p*
	*B*	*β*	S.E.	C.R.	*p*	*B*	*β*	S.E.	C.R.	*p*		
Physical activity → PSQI scores	−0.041	−0.271	0.007	−5.955	< 0.001	−0.046	−0.266	0.007	−6.710	< 0.001	0.505	0.614
Negative attentional bias → PSQI scores	2.021	0.302	0.318	6.35	< 0.001	1.706	0.226	0.322	5.304	< 0.001	0.696	0.487
Bedtime procrastination → PSQI scores	0.788	0.123	0.293	2.687	0.007	2.321	0.341	0.29	8.008	< 0.001	−3.719	< 0.001
Physical activity → Negative attentional bias	−0.007	−0.326	0.001	−6.609	< 0.001	−0.008	−0.358	0.001	−7.577	< 0.001	0.707	0.48
Physical activity → Bedtime procrastination	−0.007	−0.294	0.001	−5.785	< 0.001	−0.006	−0.249	0.001	−5.291	< 0.001	−0.707	0.48
Negative attentional bias → Bedtime procrastination	0.102	0.097	0.054	1.878	0.06	0.29	0.262	0.056	5.222	< 0.001	−2.417	0.016

B, unstandardized coefficient; *β*, standardized coefficient; SE, standard error; C.R., critical ratio; PSQI, Pittsburgh Sleep Quality Index. Higher PSQI scores indicate poorer sleep quality. Group C.R. and Group p represent between-gender differences in path coefficients.

### Comparison of indirect associations across gender

To further examine gender differences in the indirect associations, the bootstrap method was applied to compare selected direct and indirect associations across male and female groups (see Table 8). The results indicated that the direct association between physical activity and PSQI scores was significant in both gender groups, with no significant difference between groups. Regarding the simple indirect associations, the pathway through negative attentional bias was significant in both groups, with no significant difference between males and females. In contrast, the indirect association through bedtime procrastination appeared stronger in the female group, and the between-group difference was statistically significant. For the sequential association pattern, the indirect association of physical activity → negative attentional bias → bedtime procrastination → PSQI scores was significant among female students but not among male students, and the between-group difference was also statistically significant. Overall, the multi-group analysis suggests that the association structure was broadly comparable across genders, although some associations, particularly those involving bedtime procrastination, appeared stronger among female students.

**Table 8:** Bootstrap comparison of selected direct and indirect associations across gender groups.

Category	Path	Male			Female			Difference		
		*B*	95% CI	*p*	*B*	95% CI	*p*	*B*	95% CI	*p*
Direct association	Physical activity → PSQI scores	−0.041	[−0.055, −0.026]	< 0.001	−0.046	[−0.061, −0.031]	< 0.001	0.005	[−0.016, 0.026]	0.645
Simple indirect association	Physical activity → Negative attentional bias → PSQI scores	−0.015	[−0.023, −0.009]	< 0.001	−0.014	[−0.022, −0.008]	< 0.001	−0.001	[−0.011, 0.009]	0.873
Simple indirect association	Physical activity → Bedtime procrastination → PSQI scores	−0.005	[−0.011, −0.001]	0.01	−0.015	[−0.024, −0.008]	< 0.001	0.009	[0.000, 0.019]	0.047
Sequential indirect association	Physical activity → Negative attentional bias → Bedtime procrastination → PSQI scores	−0.001	[−0.002, 0.000]	0.055	−0.006	[−0.009, −0.003]	< 0.001	0.005	[0.002, 0.009]	< 0.001

B, unstandardized bootstrap estimate; CI, confidence interval; PSQI, Pittsburgh Sleep Quality Index. Difference values represent male minus female estimates. Bootstrap confidence intervals that do not include zero indicate statistically significant between-group differences.

## Discussion

### Physical activity and sleep quality: a multilevel association structure

The results of this study indicate that physical activity is significantly negatively associated with PSQI scores, reflecting a systematic association between physical activity and better sleep quality. This finding is consistent with previous research documenting a stable association between exercise and sleep quality (Dolezal et al., 2017). Existing systematic reviews and meta-analyses have shown that regular physical activity is related to better subjective sleep quality, shorter sleep onset latency, and fewer nocturnal awakenings (Kelley and Kelley, 2017). It is important to note that, given the cross-sectional design of the present study, the findings should not be interpreted as indicating that physical activity leads to improvements in sleep quality. Instead, the association observed here is better understood as a stable pattern of covariation. Individuals who engage in regular physical activity often display more structured daily routines, higher levels of self-regulation, and more positive psychological states (Zhang et al., 2024), all of which are themselves closely associated with sleep quality (Wang and Boros, 2021). From a theoretical perspective, the hyperarousal model suggests that sleep problems are related to sustained physiological and psychological activation (Riemann et al., 2010). Within this framework, physical activity may be associated with an individual’s broader arousal regulation patterns. For example, individuals with higher levels of physical activity may exhibit more balanced autonomic nervous system functioning or more stable circadian rhythms (Buijs et al., 2013; Yamanaka et al., 2015). Although this interpretation remains theoretical, it provides a plausible framework for understanding the present findings. Therefore, in the current study, physical activity is better conceptualized as a key component within a broader cluster of health behaviors, rather than as an isolated causal factor. It should be noted that although the observed associations were statistically significant, their magnitudes were relatively modest, and statistical significance does not necessarily imply strong practical impact. This suggests that sleep quality is likely influenced by multiple factors beyond those included in the present model.

### Negative attentional bias: cognitive processing and sleep quality

The results further indicate that negative attentional bias shows a significant indirect association in the relationship between physical activity and PSQI scores. Specifically, higher levels of physical activity were associated with lower levels of negative attentional bias, whereas higher levels of negative attentional bias were associated with poorer sleep quality. Within the framework of cognitive emotion regulation theory, attentional bias is often considered a relatively stable tendency in information processing (Todd et al., 2012; Van Bockstaele et al., 2019). Previous studies have shown that selective attention to negative information is closely related to persistent negative emotional experiences (Bastian et al., 2017), which may prolong cognitive activation and be associated with difficulties in falling asleep (Gobin et al., 2015). In addition, experimental research suggests that sleep deprivation itself can increase sensitivity to negative stimuli (Palmer et al., 2024), indicating that attentional bias and sleep quality may be bidirectionally related. Therefore, the pattern observed in the present study may reflect a covariation structure rather than a strictly unidirectional pathway. In the field of exercise psychology, some studies have reported that participation in physical activity is positively associated with executive control ability and attentional flexibility (Salas-Gomez et al., 2020). These findings provide indirect support for the association structure linking physical activity, attentional processing, and sleep quality. In this context, negative attentional bias may be better understood as a cognitive risk marker, rather than an independent determining factor of sleep outcomes.

### Bedtime procrastination: a behavioral regulation pattern

The present study also found that bedtime procrastination showed a significant indirect association in the relationship between physical activity and PSQI scores. Higher levels of physical activity were associated with lower levels of bedtime procrastination, whereas higher bedtime procrastination was associated with poorer sleep quality. According to self-regulation theory, bedtime procrastination can be understood as a behavioral tendency in which short-term motivational preferences override long-term health goals (Kadzikowska-Wrzosek, 2018). Previous research has shown that bedtime procrastination is significantly associated with reduced self-control, emotional exhaustion, and poorer time management abilities (Hamvai et al., 2023). In addition, higher levels of bedtime procrastination have been consistently linked to shorter sleep duration and poorer sleep quality (Uygur and Bahar, 2023). Within the field of health behavior research, physical activity is often viewed as an important behavioral indicator of self-regulation capacity (Faílde-Garrido et al., 2022). Individuals who maintain higher levels of physical activity tend to demonstrate greater behavioral discipline and more regular daily routines (Yu et al., 2022). From this perspective, the association pattern observed in the present study (physical activity, bedtime procrastination, and sleep quality)—may reflect a broader covariation structure of self-regulatory capacity. It is also worth noting that some studies suggest that sleep deprivation itself may impair self-control, which may also be associated with higher levels of procrastination behaviors (Gorgol-Waleriańczyk and Nowosadko, 2026). Therefore, the associations observed in this study may reflect a cyclical or mutually reinforcing pattern rather than a strictly linear process.

### Cognitive–behavioral sequential association

The analysis indicated that physical activity, negative attentional bias, and bedtime procrastination were involved in a sequential indirect association pattern related to PSQI scores. The sequential indirect association accounted for 4.2% of the total association, indicating that this pathway represented a relatively small component of the overall association structure. Nevertheless, it may still help describe potential connections across different psychological levels. Within cognitive–behavioral integrative models, patterns of information processing are considered to be associated with individuals’ emotional responses and behavioral decision tendencies (Brandt et al., 2025). Individuals who show a stronger tendency to attend to negative information may be associated with greater emotional exhaustion or heightened psychological stress, which may be associated with a greater tendency to engage in evening entertainment activities or procrastination behaviors as a form of emotion regulation. Related cognitive–behavioral association patterns have also been discussed in sleep research (Kroese et al., 2016). However, it should be emphasized that the cross-sectional data used in the present study do not allow for verification of temporal order. Therefore, the sequential association pattern identified here is better interpreted as a structural pattern of covariation, rather than a strict causal chain.

### Gender differences: variations in path strength rather than structural differences

The multi-group structural equation modeling results showed that the overall path structure was largely consistent across male and female samples, although some path coefficients differed significantly. These results suggest that the association structure linking physical activity, cognitive processing, behavioral regulation, and sleep quality may be broadly comparable across gender groups, while the strength of specific associations may vary between genders. Previous research has shown that women tend to exhibit higher sensitivity in emotional processing, stronger tendencies toward rumination, and greater stress reactivity compared with men (Nolen-Hoeksema, 2012). In addition, women generally report higher prevalence rates of sleep problems than men (Madrid-Valero et al., 2017). From this perspective, the observed differences in path strength may be related to gender differences in emotional regulation sensitivity, although this interpretation remains speculative because emotional sensitivity and rumination were not directly measured in the present study. Another point worth considering is that the broad comparability of the model across gender groups suggests that the cognitive–behavioral sequential association pattern may represent a similar association structure in male and female students. This finding may have theoretical implications, as it suggests that the psychological association structure underlying sleep-related processes may follow a broadly similar pattern across gender groups, while gender differences may influence the magnitude of some associations. At the same time, caution is needed to avoid overinterpretation. Gender differences may also be influenced by social role expectations, behavioral habits, or sample composition. Therefore, future research should further examine measurement invariance and structural invariance across gender groups, in order to more rigorously determine the role and boundaries of gender-related effects.

### Theoretical implications and model reflection

The theoretical contribution of the present study lies not in introducing entirely new variables, but in refining how existing constructs are integrated within a unified association-based analytical framework. Previous research has typically relied on single-variable explanatory models or focused on relatively homogeneous pathways, most commonly emphasizing emotional processes. Even in studies adopting multi-step indirect association models, the focus has largely remained on emotional or stress-related pathways, with limited consideration of how different levels of psychological processes may be related. In contrast, the present study builds on this line of research by integrating cognitive processing (negative attentional bias) and behavioral regulation (bedtime procrastination) within the same model. Rather than examining these factors in isolation, the current framework highlights their potential structural associations, suggesting that associations between physical activity and sleep quality may be reflected across multiple psychological levels. Compared with prior multi-step studies that primarily emphasize emotional pathways (e.g., resilience and emotional distress), the present study offers a complementary perspective by incorporating both cognitive and behavioral components into a single analytical framework. This multi-level integration allows for a more nuanced understanding of how health-related behaviors may be associated with sleep-related outcomes through related patterns of psychological functioning. Importantly, the contribution of this study should be understood in terms of structural integration rather than causal explanation. By identifying a patterned association across cognitive and behavioral domains, the findings offer a more differentiated framework for interpreting the associations among physical activity, psychological processes, and sleep quality.

## Limitations

This study has several limitations that should be noted. First, due to the cross-sectional design, temporal ordering among variables cannot be established, and the observed associations should be interpreted as patterns of statistical covariation rather than causal pathways. Second, physical activity was assessed using a brief three-item self-report measure (PARS-3), which may be subject to recall bias, reporting bias, and subjective interpretation of exercise intensity. The PARS-3 provides a composite index of physical activity based on intensity, duration, and frequency, but it does not distinguish between different types of physical activity, such as aerobic or resistance exercise, nor does it assess the time of day at which physical activity occurs. These details may be relevant to sleep-related outcomes and should be considered in future studies. Third, the sample was drawn from four universities in East China using class-based cluster sampling, which may limit the generalizability of the findings and should be considered when interpreting the results. Future research using cluster sampling may further consider class- or university-level clustering when relevant identifiers are available. Fourth, because all variables were measured using self-report questionnaires, common method bias may still be present despite the results of Harman’s single-factor test. Fifth, the present study did not include several sleep-related and health-related covariates, such as body mass index, smoking, alcohol and caffeine consumption, chronic health conditions, screen use, grade level, psychological distress, and other sleep-related behaviors. Future studies are encouraged to employ longitudinal or experimental designs, include more diverse samples and covariates, and use objective or multi-method assessments to further examine the observed associations.

## Conclusion

The present study examined the association between physical activity and PSQI-assessed sleep quality among college students, with attention to the sequential indirect associations of negative attentional bias and bedtime procrastination. The findings suggest that higher levels of physical activity were associated with lower PSQI scores, lower negative attentional bias, and lower bedtime procrastination. Negative attentional bias and bedtime procrastination were also involved in simple and sequential indirect association patterns linking physical activity and sleep quality. Multi-group analyses indicated that the overall association structure was broadly comparable across gender groups, although associations involving bedtime procrastination appeared stronger among female students. Given the cross-sectional design, these findings should be interpreted as patterns of statistical association rather than causal pathways. Future longitudinal and experimental studies are needed to further examine the temporal and potentially bidirectional relationships among physical activity, cognitive processing, behavioral regulation, and sleep quality.

## Data Availability

The de-identified data supporting the findings of this study are available from the first author upon reasonable request, subject to applicable ethical and privacy considerations.
